# Can Routine Commercial Cord Blood Banking Be Scientifically and Ethically Justified?

**DOI:** 10.1371/journal.pmed.0020044

**Published:** 2005-02-22

**Authors:** Nicholas M Fisk, Irene A. G Roberts, Roger Markwald, Vladimir Mironov

## Abstract

Background to the debate: Umbilical cord blood—the blood that remains in the placenta after birth—can be collected and stored frozen for years. A well-accepted use of cord blood is as an alternative to bone marrow as a source of hematopoietic stem cells for allogeneic transplantation to siblings or to unrelated recipients; women can donate cord blood for unrelated recipients to public banks. However, private banks are now open that offer expectant parents the option to pay a fee for the chance to store cord blood for possible future use by that same child (autologous transplantation.)

## Abstract 2

Private banks offer expectant parents the option to pay a fee for the chance to store cord blood for possible future use by the child. The practice is controversial, for scientific and ethical reasons

## Nicholas Fisk and Irene Roberts's Viewpoint: There Are Good Reasons to Be Wary of Private Banking

No one disputes the merit of public cord blood banking, in which women altruistically donate umbilical cord blood (UCB) for haemopoietic stem cell (HSC) transplantation, in a way similar to bone marrow donation. Unrelated UCB transplants have good outcomes in children and are associated with less graft-versus-host disease than adult marrow or peripheral blood stem cells [1,2]. Public cord blood banks also increase the availability of donor HSCs for ethnic groups underrepresented in bone marrow registries [3]. Similarly, there is little argument against storing UCB from siblings in families with a known genetic disease amenable to HSC transplantation [4].

The validity of directed UCB storage in “low risk” families, however, has been widely challenged. After early concerns from the American Academy of Pediatrics [5] and American College of Obstetricians and Gynecologists [6], the United Kingdom's Royal College of Obstetricians and Gynaecologists concluded in 2001 that routine, directed commercial UCB storage could not be justified scientifically, was logistically difficult, and therefore could not be recommended [7]. In 2002, the French National Consultative Ethics Committee for Health and Life Sciences reached similar conclusions [8]. In Italy the practice has been banned. A recent European Union report highlighted serious ethical concerns about commercial UCB banks and questioned their legitimacy in selling a service of no real use [9]. So what's wrong with allowing parents who can afford it the biological luxury of storing their child's stem cells?[Fig pmed-0020044-g001]

**Figure pmed-0020044-g001:**
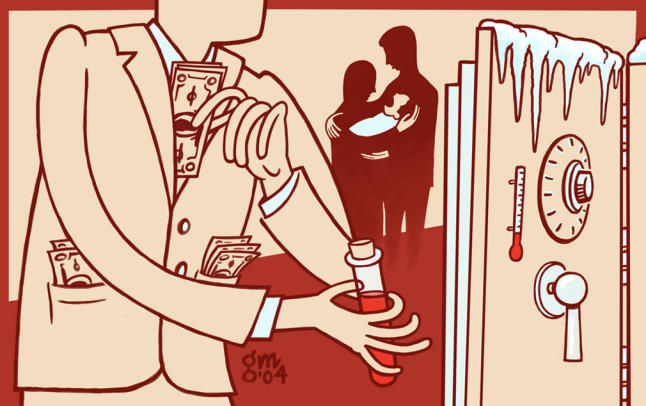
Are commercial UCB banks exploiting the emotional vulnerabilities of parents for financial gain? (Illustration: Giovanni Maki)

First, UCB is very unlikely ever to be used. The probability of needing an autologous transplant is less than one in 20,000 [9,10], although commercial providers quote figures at least an order of magnitude higher, often confusing prearranged usage in at risk children with unanticipated use in those at low risk. For acute leukaemia, perhaps the most likely indication for autologous UCB transplantation, improvements in conventional therapy and allogeneic transplantation mean few proceed to autologous transplantation. In any case, there are arguments against the use of autologous UCB, including the presence of pre-leukaemic mutations and the high rate of relapse [11]. Similar considerations apply to bone marrow failure [11]. Of current indications for HSC transplantation [12], only for solid tumours, lymphomas, and auto-immune disorders might autologous UCB find a role, and even here, UCB collections often contain only enough HSCs to reconstitute children (not adults). Other uses for UCB remain speculative since it is unclear whether non-haemopoietic stem cells are present in sufficient numbers for use against degenerative conditions. Even in the uncommon event of a requirement for autologous stem cells, failure to store UCB is unlikely to be disastrous; HSCs could still be harvested from bone marrow or peripheral blood, and multipotent stem cells are increasingly being isolated from other accessible sources (e.g., deciduous teeth).

Umbilical cord blood is very unlikely ever to be used.

Second, there are important moral issues. The persuasive promotional materials of commercial UCB banks target parents at a vulnerable time, urging them to take this “once in a lifetime opportunity” to “save the key components to future medical treatment” and freeze “a spare immune system” [7]. Even at a typical cost of several thousand dollars, how could any responsible parent fail to provide for their child's future by preserving “something that may conceivably save his or her life”? As well as enumerating conditions currently treated by HSC transplantation, such literature boasts lengthy lists of diseases potentially amenable to stem cell therapy in the future, including Parkinson disease, diabetes, cancer, and heart disease. Such banks have been said to raise hopes of utopia and to use the promise of “helping children” to disguise a mercantile project[Fig pmed-0020044-g002]

**Figure pmed-0020044-g002:**
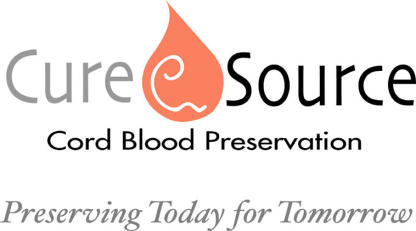
CureSource, a commercial UCB bank, believes that banking is a “once in a lifetime opportunity” (Figure: CureSource)

Third, collection imposes a considerable logistic burden on the obstetrician or midwife. In addition to consent, parental blood collection, and the associated packaging and paperwork, a large volume of blood has to be collected from the umbilical vessels in utero, requiring multiple syringes under aseptic technique. This may distract professionals from their primary task of caring for the mother and baby at this risky time or, more generally, divert delivery room staff from attending others [7]. This applies particularly in multiple or operative deliveries, and thus UCB collection is not recommended at complicated births [5]. These problems do not apply to altruistic donations to public cord blood banks, which can be harvested less intrusively ex utero; inadequate or logistically difficult samples can be discarded or forgone without consequence [3].

Finally, individual UCB banks need to remain in business long term if cryopreserved stem cells are to be retrieved. The commercial attractiveness of a service paid years in advance is attested to by the burgeoning number of private providers, yet it seems unlikely that all will survive. Indeed, some US providers are already in trouble for infringing collection patents. There remain reservations about whether laboratories will meet national standards and be accredited. There is a further danger that misplaced enthusiasm for commercial auto-collection will undermine the proven utility of altruistic public cord blood banks.

Notwithstanding the above, we accept that the utility of UCB storage in low-risk families is very different from the entirely speculative post-mortem cryonics industry. We acknowledge the possibility that autologously stored UCB stem cells may eventually be used. Indeed, recent research documenting the multi-potentiality of UCB mesenchymal lineages [13] and the in vitro expandability of cord HSC numbers sufficient to transplant an adult [14,15] may even improve such prospects. Private banks, however, must provide clear, honest, and unambiguous information for their customers. EU guidance recommends they be told that the likelihood of stored UCB stem cells being used to treat their child is negligible and that future therapeutic possibilities are very hypothetical [9].

## Roger Markwald and Vladimir Mironov's Viewpoint: No One Has a Second Chance to Collect Their Cord Blood

Stem cells may potentially be used in life-saving therapies for degenerative diseases or injuries. Stem cells self-replicate and are multi-potential—they can differentiate into diverse cell types [13]. While stem cells can come from many sources, our viewpoint is that UCB is an important source of progenitor (stem) cells that can be used as an immediate alternative for bone marrow transplantation and for engineering healthy new cells and tissues.

To fully realize this potential will require collection and banking of UCB cells, which are harvested without pain or trauma from placental structures that are normally discarded after birth. We realize that UCB banking (public and private) has sparked controversy. Critics of routine banking question its cost-to-benefit ratio, citing doubts about the clinical relevance of cord stem cells or the likelihood that they will ever be used [16]. Other critics argue that embryonic stem cells (ESCs) are the better option.

The “stemness” of UCB cells is not merely theoretical (as suggested by Steinbrook; [16]). UCB has two types of multi-potential progenitor cells—HSCs and mesenchymal stem cells. These express different cell surface markers, making it possible to show that HSCs can differentiate into new red and white blood cells and, as with mesenchymal stem cells, can also transdifferentiate in vivo into liver, kidney, brain, bone, skeletal, and cardiac muscle cells [13,17,18,19,20]. While ESCs have the potential to form all types of cells, they are harvested from embryos shortly after fertilisation, raising moral and legal issues not attributed to UCB cells [21].

The real question is who should pay for umbilical cord blood collection and storage.

ESCs also represent an allogeneic source of cells—they are derived from another individual whose tissue type does not match up with the recipient, resulting in immune rejection when transplanted [22]. We know of no clinical or preclinical animal study that provides hard evidence of functional integration (without immune rejection) of transplanted ESCs. Even with somatic nuclear transfer (cloning), ESCs remain allogeneic, as they still have foreign mitochondrial DNA for which there remains untested potential risk for auto-immune diseases. In contrast, 2,000 allogeneic UCB transplants have been performed, mostly in children, for the treatment of a variety of malignant and nonmalignant conditions [22]. A London Cord Blood Bank report found that two years after transplantation the survival rate varied between 54% and 69%, depending upon the number of matched units [23]. For reasons not fully understood, allogeneically transplanted UCB cells have immune tolerance (of HLA mismatch) [24], and the risk of causing graft-versus-host disease is considered to be acceptable [24,25].

With millions of healthy babies born each year, there is potentially a large UCB supply that can be stored, tissue-typed, and made available at short notice. If saved for potential use by the donor, UCB cells become a source of perfectly matched, autologous stem cells (plus there is a 25% probability of being an exact match for a sibling). Yet the American Academy of Pediatrics came out against UCB banking, saying that the odds (with some exceptions) of a donor ever using a UCB sample were low, between 1/1,000 and 1/200,000. While the chance of a donor benefiting may presently be low, this does not automatically mean that another member of society could not benefit. For people with genetic diseases or cancers, the chances of finding an immune-tolerant donor match would obviously be increased by the expansion of cord blood sampling. Also, at the pace that stem cell research is moving, perhaps there will be new uses for UCB cells in the next decade, especially in the field of tissue engineering [26]. Importantly, unlike bone marrow, an increase in UCB samples will enhance availability for every ethnic group for tissue matching. What is certain is that no one has a second chance to collect their cord blood.

Who should operate cord blood banks—the private or the public sector? There are around 20 private UCB banks in the United States. They charge a collection fee, typically $1,000–$1,500, which includes testing for pathogens and genotyping. Samples are maintained in a frozen state for around $100 a year. An additional $15,000–$25,000 is charged if a sample is used for transplantation (usually covered by health insurance). The cost of UCB cell transplantation is significantly less than bone marrow transplantation, and the risk of graft-versus-host disease is lower [24]. The private sector, not government, has been the innovator for most new technology related to harvesting, storing, and utilizing cord blood as well as stem cell research. Licensing fees and patent protection are essential to biotechnology companies—they are needed to attract venture capital, build businesses, and develop new technologies. The only alternative in most countries is public cord banks, which suffer from insufficient funding.

Any exploitation by companies of the vulnerabilities of expectant parents for financial gain is clearly unacceptable. Federal legislation to establish a national cord blood stem cell bank network—free to all donors—has been introduced in the US Senate and House of Representatives that, if approved, should diminish the risk of exploitation. But unless the network is well-designed from a sociological viewpoint, it could generate a situation where not all cultural and ethnic groups are represented or where benefits are accessible only to families with health insurance or sufficient income to afford transplants. It still remains difficult to find full matches for African, Asian, and Native Americans—mostly because of an insufficient number of UCB donors and the diversity of HLA types in different ethnic groups [16].

The real question is who should pay for UCB collection and storage—the individual donor, who currently has only a small prospect of using their cord blood, or society as a whole? We believe that it is the job of government to assure that people of all ethnic groups are informed and educated about donating UCB. Then, to facilitate UCB banking and the development of technological innovations for its storage and clinical utility, we recommend a national network that is a mixture of for profit, non-profit, and governmental organizations.

## Fisk and Roberts's Response to Markwald and Mironov's Viewpoint

Markwald and Mironov argue that commercial UCB banking is ethically justified on the grounds that UCB transplantation is effective treatment for many haematological disorders, that autologous UCB is a useful future source of stem cells for the donor, and that there is no second chance to collect these cells.

We did not dispute (indeed we acknowledged) the value of UCB HSCs for the treatment of many malignant and non-malignant haematological disorders. However, evidence of their value derives from allogeneic HSCs from public UCB banks [27]. Like many in the routine UCB collection industry, Markwald and Mironov fail completely to distinguish between public and private banks in their discussion, and further neglect to mention that most transplants have been of allogeneic cells donated altruistically by non-related donor families.

Markwald and Mironov state that the real question is who should pay for routine UCB collection and storage. However, they take no account of the considerable logistic burden this imposes, the extremely low chance that autologous cells will ever be used (less than one in 20,000), and the costs of routine UCB collection [9]. They also fail to mention that autologous UCB HSCs are frequently unsuitable for use for two reasons. First, they cannot cure inherited disorders (e.g., β-thalassaemia major or congenital bone marrow failure syndromes), and second, clinically hidden pre-leukaemic and/or leukaemic cells may be present in UCB at birth in children who years later develop full-blown leukaemia [28]. In addition, the authors introduce the irrelevant argument of the likely unsuitability of ESC transplants, with which, given the propensity of these transplants to cause teratomas, we agree [29].

Thus the real questions are, first, why should society in general, or the government as a representative of at least a substantial proportion of society, pay for a service not shown to be of any real use? (After all, as we pointed out, autologous HSCs are rarely required and there is no evidence that UCB can treat degenerative disease in elderly humans.) And second, why should commercial banks be allowed to continue to target vulnerable parents anxious to do the best for their children while making no mention of the low chance of use, of alternative sources of available stem cells (e.g., autologous marrow, a better source of non-haemopoietic stem cells), or of the risks of reducing stocks of allogeneic HSC in public UCB banks?

## Markwald and Mironov's Response to Fisk and Roberts's Viewpoint

We agree with Fisk and Roberts that exploiting the emotional vulnerabilities of expectant parents is unjustifiable—thus we support regulation of UCB banking, monitoring, certification, and informed consent. But we disagree that there is a lack of solid scientific evidence for UCB collection and that “future therapeutic possibilities are very hypothetical.” Research on stem cells is advancing rapidly, and stem cells derived from UCB are emerging as a reasonable first choice for the field of regenerative medicine.

Fisk and Roberts are inconsistent in their views. They claim that stem cells collected in UCB units often are not “in sufficient numbers for use against degenerative conditions” in adult life but then acknowledge that “the in vitro expandability of cord HSC numbers is sufficient for transplantation into an adult.” They argue that private and public UCB collections create dramatically different “logistic burdens,” but in our experience, the syringes, paperwork, and level of personal distraction are generally the same for public or private banking.

We strongly disagree that private UCB banking has no future. While we anticipate a consolidation phase for this industry, surviving companies should be eager to acquire UCB units collected from competitors. If all stem cell sources were under a state monopoly—without private sector contribution—there would be less incentive or opportunity for fostering innovation in long-term storage, expansion, or phenotype characterization of UCB stem cells. The growth of new biotech companies focused on regenerative medicine would be discouraged, compromised, or undermined by the absence of competition, inadequate access to venture capital, and the typical resistance of state health-care systems and their affiliated medical professionals to innovation.

Fisk and Roberts are creating obfuscations by mixing “speculative cryobionic companies” that promise “immortality and eternity” with serious biotech companies and private UCB banks that focus on a realistic commercialisation of UCB stem cells as a platform for promoting new biotech initiatives.

The collection and storage of UCB stem cells is an opportunity for society to build a representative collection of UCB units that can improve the chances of identifying suitably matched donors for transplantation. Human ESCs are mired in ethical concerns and concerns about immunological intolerance. Autologous cells from the bone marrow or elsewhere lose their attractiveness if there is a genetic mutation or a progressive loss of “stemness” due to normal aging [30]. UCB cells offer the best short- and long-term hope for treating sick children with cancers or adults with a variety of diseased organs and tissues.

## Abbreviations

ESC: embryonic stem cell
HSC: haemopoietic stem cell
UCB: umbilical cord blood
